# The neuroscientific study of spiritual practices

**DOI:** 10.3389/fpsyg.2014.00215

**Published:** 2014-03-18

**Authors:** Andrew B. Newberg

**Affiliations:** Myrna Brind Center of Integrative Medicine, Thomas Jefferson UniversityPhiladelphia, PA, USA

**Keywords:** spiritual practice, meditation, prayer, neuroimaging, physiology, methodology

## Abstract

The purpose of this paper will be to provide a perspective on the current state of the research evaluating the neurobiological correlates of spiritual practices and review the methodological issues that confront this research field. There are many types of spiritual practices that might be studied including prayer and meditation, as well as unusual practices such as mediumistic trance states, speaking in tongues, and also drug-induced experiences. Current studies have utilized neuroimaging techniques including functional magnetic resonance imaging, single photon emission computed tomography, and positron emission tomography. These studies have helped elucidate the neurobiological mechanisms associated with spiritual practices. Such studies confront unique challenges for scientific methodology including determining the most appropriate objective measures such as neuroimaging studies and physiological parameters, and correlating them with subjective measures that help capture states of spiritual significance. Overall, a neuroscientific study of spiritual practices and experiences has the potential to provide fascinating data to further our understanding of the relationship between the brain and such phenomena.

## INTRODUCTION

The recently expanding field of research exploring the neuroscience of religious and spiritual practices and associated experiences has raised important issues regarding the validity, importance, relevance, and need for such research. At the outset, it should be stated that the focus of this paper is specifically on practices that have a spiritual or religious context such as prayer, speaking in tongues, or certain types of meditation. However, it should also be noted that there are a substantial number of studies that have evaluated meditation practices that are not specifically spiritual (i.e., secular mindfulness programs). There are fewer studies on specifically spiritual practices. Studies of meditation practices not related to a particular religious or spiritual tradition still provide information that may contribute to the overall study of religious and spiritual phenomena, but that is not the focus of this paper. The best way to develop this field is to determine the methodological issues that currently affect the field and explore how best to address such issues so that future investigations can be as robust as possible. This paper will review four components of this area of research with a critical perspective on methodology and analysis: (1) appropriate measures and definitions; (2) subject selection and comparison groups; (3) study design; and (4) theological and epistemological perspectives.

## MEASUREMENT AND DEFINITION OF SPIRITUALITY AND RELIGIOUSNESS

### DEFINITIONS

As someone who has studied the neurophysiology of a variety of practices with both spiritual and non-spiritual goals, I have realized that great importance and complexity in first trying to define spirituality and religiousness as terms and also to differentiate spiritual experiences from spiritual practices (Newberg, 2010). This latter issue has significant implications for research as the practices might be studied using different approaches from the experiences (Nash and Newberg, 2013). Although a variety of approaches may be used, for an adequate development of this field, it is important to define terms operationally so that they can be studied effectively. There is significant overlap between these terms with both having personal and group elements, emotional and cognitive elements, and experiential elements. One of the primary distinctions is that religiousness tends to relate to a particular religious tradition. Perhaps the most important point to be made is that every researcher or scholar should provide in their writings the definition of religiousness or spirituality that they applied so that others can better assess the usefulness of their scholarship.

### SUBJECTIVE MEASURES

In some sense, the most relevant measures of religious and spiritual practices are those that relate to the subjective nature of their associated experiences. When a person has a religious or spiritual experience, it is frequently described in terms of cognitive, behavior, and emotional parameters. Importantly, a person will label or define the experience as “spiritual” which differentiates the experience from others which are regarded as “non-spiritual.” The issue with measuring the subjective elements of these phenomena is crucial since these elements are not immediately observable by an outside investigator. The problem becomes more difficult when comparing experiences across individuals and cultures. The question for any researcher is how to get some handle on the subjective component of such experiences. Is there a way to quantify and compare these subjective feelings and thoughts individuals have regarding their spiritual experiences? And how does a researcher evaluate the authenticity of a “religious or spiritual” experience? For example, some researchers suggest that the concept of “certainty” or “meaningfulness” is an important element of spiritual practices and experiences. Other scholars have explored the relationship between meaningfulness, social connectedness, and love (Frederickson, 2013), all of which can also be tied to specific areas of the brain.

A number of attempts have developed self-reporting scales that measure the subjective nature of a particular religious or spiritual elements. The book, Measures of Religiosity (Hill and Hood, 1999) provides an extensive review of various scales and questionnaires that assess everything from feelings of religious commitment, to mystical experiences, to the direct apprehension of God. Some have been assessed for validity and reliability which is critical if these scales are to have any use in future research studies. Of course, defining what a spiritual experience is, while of critical importance, proves to be highly problematic from a scientific perspective. If someone defines spiritual as a feeling of “awe” and another as a feeling of “oneness,” what types of questions should be used to assess and measure spirituality? Most scales of spirituality and religiousness require the individual to respond in terms of psychological, affective, or cognitive elements. Such measures are quite valuable for exploring the neural correlates of spiritual experiences because psychological, affective, and cognitive elements can usually be related to specific brain structures or functions. However, a problem with phrasing questions in this way is that one may not get at something that might be “truly” spiritual. Finally, it is unclear how science should handle what are frequently referred to as anomalous elements of such experiences. These elements include near death experiences in which people purportedly describe the environment around them at the time of death, or mediums who perceive encounters with different spirits (Beauregard et al., 2009; Peres et al., 2012). Such anomalous events are important for science to consider, but certainly pose a substantial challenge as well.

## OBJECTIVE MEASURES OF SPIRITUALITY

Objective measures of religious and spiritual phenomena that pertain to the neurosciences include a variety of physiological and neurobiological measures. Most studies to date have been performed on meditation practices that are not specifically spiritual in nature, but the methods and results from these studies should inform future studies of spiritual practices. A number of studies have revealed changes in blood pressure and heart rate associated with meditation based practices, although these practices were not associated with spiritual approaches (Sudsuang et al., 1991; Jevning et al., 1992; Koenig et al., 2001). The autonomic nervous system changes may be complex involving both a relaxation and also an arousal response (Hugdahl, 1996). Several studies have reported predominant parasympathetic activity during spiritual practices which includes decreased heart rate and blood pressure, decreased respiratory rate, and decreased oxygen metabolism (Sudsuang et al., 1991; Jevning et al., 1992; Bernardi et al., 2001; Travis, 2001). However, other studies have suggested a mutual activation of parasympathetic and sympathetic systems by demonstrating an increase in the variability of heart rate during meditation (Peng et al., 1999). Measures of hormone and immune function have also been explored especially as an adjunct measure to various clinical outcomes (O’Halloran et al., 1985; Walton et al., 1995; Tooley et al., 2000; Infante et al., 2001). Hormonal changes associated with spiritual practices include those to cortisol, noradrenaline, endorphins, sex hormones, and growth hormone (Werner et al., 1986; MacLean et al., 1997; Nidhi et al., 2013; Sooksawat et al., 2013). Again though, these practices were not specifically spiritual. There are only a few report of physiological changes associated with specifically spiritual practices such as the Rosary.

Neurobiological changes associated with religious and spiritual practices can be observed through a number of techniques that each have their own advantages and disadvantages. Early studies of meditation practices often used electroencephalography (EEG) which measures the electrical activity in the brain (Banquet, 1973; Hirai, 1974; Hebert and Lehmann, 1977; Corby et al., 1978). EEG is valuable because it is relatively non-invasive and has very good temporal resolution, although it can suffer from artifact related to the skull or scalp. Overall, EEG has continued to be useful in the evaluation of specific meditation states (Lehmann et al., 2001; Aftanas and Golocheikine, 2002; Travis and Arenander, 2004). The major problem with EEG is low spatial resolution which can only be localized over very broad areas of the brain. A newer technique, with improved spatial resolution, called magnetoencephalography (MEG) has been developed and already used to explore meditation practices (Yamamoto et al., 2006; Kerr et al., 2013).

Functional neuroimaging studies of religious and spiritual practices have utilized positron emission tomography (PET), single photon emission computed tomography (SPECT), and functional magnetic resonance imaging (fMRI). Each technique has its advantages and limitations with respect to evaluating religious and spiritual phenomena. Functional MRI primarily measures changes in cerebral blood flow in brain structures during a specific task. Functional MRI has very good spatial resolution and also has excellent temporal resolution, although not as good as EEG or MEG. For example, fMRI can evaluate the differences in blood flow between 10 different prayer states in one imaging session over a period of 20 min. Furthermore, fMRI does not involve any radioactive exposure. The disadvantages are that images must be obtained while the subject is lying in the scanner which can make up to 100 decibels of noise. This can be distracting when individual are performing spiritual practices and also prevents studying practices that require certain postures or movements. However, several investigators, including our group, have successfully utilized fMRI for the study of meditation (Lazar et al., 2000; Wang et al., 2011; Vago and Silbersweig, 2012). A final disadvantage is that at the present moment, fMRI cannot be used to evaluate neurotransmitter systems which might ultimately be of interest in the study of spiritual practices. However, one study did utilize magnetic resonance spectroscopy to find an increase in gamma amino butyric acid during yoga training (Streeter et al., 2007).

Positron emission tomography imaging has relatively good spatial resolution (comparable to fMRI) but SPECT is slightly worse. PET and SPECT both require the injection of a radioactive tracer which results in some radiation exposure, although this is usually low. Depending on the tracer utilized, a variety of neurobiological parameters can be measured including cerebral blood flow, metabolism, and different neurotransmitters. The ability to measure neurotransmitter systems is unique to PET and SPECT imaging at the present time. Some of the more common tracers can be injected through an existing intravenous catheter when the subject is not in the scanner allowing for a more favorable environment for performing spiritual practices (Herzog et al., 1990–1991; Newberg et al., 2001). A major disadvantage to PET and SPECT imaging is that these techniques have generally poor temporal resolution. Depending on the tracer, this resolution can be as good as several minutes and as bad as several hours or even days. Usually only two or three states might be measured in the same imaging session if the appropriate radiopharmaceutical is used (Lou et al., 1999; Newberg et al., 2006). Since spiritual experiences may be quite brief, it is not clear how effectively neuroimaging studies might be able to capture the specific moment related to something spiritual.

In spite of these and other technical limitations, neuroimaging studies have been successfully utilized to evaluate specific spiritual and meditative practices. There are a growing number of studies which have spanned the different neuroimaging techniques (Lou et al., 1999; Newberg et al., 2001, 2003; Kjaer et al., 2002; Lutz et al., 2008; Brewer et al., 2011; Xue et al., 2011). Interestingly, there appears to be some coherence of their findings with the frontal lobes, parietal lobes, thalamus, and limbic system frequently related in a network associated with such practices. However, different practices also yield distinct brain function patterns. For example, meditation practices often demonstrate increased frontal lobe function while trance practices often demonstrate decreased frontal lobe function (Herzog et al., 1990–1991; Lazar et al., 2000; Newberg et al., 2001; Tang et al., 2009; Peres et al., 2012). Future studies will certainly be necessary to more thoroughly evaluate the neurobiological changes that occur in the brain during various religious and spiritual phenomena. Ultimately, such studies should also attempt to integrate neurological changes with body physiological effects as there are a number of studies which have demonstrated a neurovisceral response between the body and various emotions and thoughts (Thayer and Lane, 2000).

## SUBJECT SELECTION AND COMPARISON GROUPS

An interesting methodological issue in the study of religious and spiritual phenomena is determining the most appropriate subjects to study and the appropriate comparison group(s). If a researcher wanted to study the physiological effects of the Catholic ritual of the Rosary, the subject group would have to consist of individuals who actually practice the Rosary. This raises another important issue which is the level of expertise or proficiency of the study subjects. How do we know how proficient a practitioner of a particular practice actually is? Should expertise be based upon subjective measures, duration of practice, or other measures? There could be very different results between novice, experienced, and master level individuals of particular practices.

In terms of comparison states and control groups, one common approach in functional neuroimaging studies is that the individual acts as their own comparison. For example, the subject can perform two similar tasks, one with and one without spiritual meaning (Azari et al., 2001). The more global issue of comparison states is to make them as equivalent as possible to the spiritual practice regarding a wide range of elements including whether eyes are opened or closed, speaking or not speaking, listening or not listening, etc. But ultimately, the comparison state should not result in a concomitant spiritual experience. Placebo effects pose another interesting problem in the study of spirituality or religiosity, and require careful consideration when designing a study so that there is an adequate placebo or control group. It is not clear what a placebo group would represent since most people know whether or not they are actually performing a spiritual practice. Perhaps more importantly is the potential overlap between the placebo response and spiritual responses in terms of their similar elements and potential underlying brain functions (Kohls et al., 2011).

## STUDY DESIGN APPROACHES

There are at least four general neuroscientific paradigms which can readily contribute to the initial operationalization of studying spiritual experience (Larson et al., 1998). There are likely many others, but these initial four paradigms include: (1) the neurophysiology of spiritual interventions, (2) drug-induced spiritual experiences, (3) neuropathologic and psychopathologic spiritual experiences, and (4) physical and psychological therapeutic interventions.

### THE NEUROBIOLOGY OF SPIRITUAL PRACTICES

The first paradigm involves studying specific spiritual practices using subjectively derived psychological and spiritual measures which can then be compared to simultaneously derived neurobiological parameters, such as electroencephalographic activity, cerebral blood flow, cerebral metabolism, and neurotransmitter activity (Newberg and Iversen, 2003). Such measures can be performed with EEG, PET, SPECT, or MRI. Many studies have now been performed utilizing imaging to evaluate meditation (see supplementary materials), but these imaging techniques may be applied to the vast array of spiritual practices such as prayer, religious singing, rituals, etc., or while a person is reflecting on a previous spiritual experience (Beauregard et al., 2009). Body physiological parameters such as blood pressure, body temperature, heart rate, and galvanic skin responses can also be measured. Other parameters such as immunological assessments, hormonal concentrations, and autonomic activity can also be evaluated to provide a thorough analysis of the effects of spiritual practices. These physiological parameters can be correlated with experiences and also with neuroimaging measures to obtain a more thorough analysis of the overall effects of spiritual practices. Further, physiological measures might yield interesting results and point to the directionality of functional changes in the brain and body associated with such practices.

### DRUG-INDUCED SPIRITUAL EXPERIENCES

A second paradigm that might be employed utilizes hallucinogenic agents that are known to result in intense spiritual experiences. Since it has long been observed that drugs taken recreationally such as opiates, LSD, and stimulants can sometimes induce spiritual-like experiences; and specific traditions use exogenous substances to induce spiritual states and experiences; careful studies of these drug-induced experiences, perhaps utilizing modern imaging techniques, may help elucidate which neurobiological mechanisms are similarly involved in more “naturally derived” spiritual experiences (i.e., through prayer or ritual). Some neuroimaging studies exploring hallucinogenic agents have already been performed (Vollenweider et al., 1997, 1999, 2000), but a more extensive study of such agents, particularly in relation to religious and spiritual experiences is required. There are obvious ethical and legal considerations with these studies, however, such studies provide fascinating perspectives on the relationship between various neurotransmitter systems affected by these drugs and religious and spiritual experiences. Further, there is growing evidence that hallucinogenic drugs might be useful in the management of addiction disorders which might help relate spirituality to the neuronal circuits associated with addition (MacLean et al., 2011).

### NEUROPATHOLOGIC AND PSYCHOPATHOLOGIC SPIRITUAL EXPERIENCES

A third paradigm would utilize patients with various neurological and/or psychological conditions. Neurological conditions including temporal lobe seizures, brain tumors, and stroke, have been associated with spiritual experiences or alterations in religious beliefs. Temporal lobe epilepsy has been associated with hyperreligiosity and religious conversions (Bear and Fedio, 1977; Bear, 1979). Head injury in the parietal lobes has been associated with feelings of self-transcendence (Urgesi et al., 2010). Psychiatric disorders such as schizophrenia and mania also have been associated with spiritual experiences and religious conversions. Delineating the type and location of pathology will aid in determining the neurobiological substrates or networks related to spiritual experiences. However, care must be taken to avoid referring to spiritual experience only in pathological terms as well as not reducing spiritual experiences only to pathophysiological mechanisms (Newberg and Lee, 2005).

### PHYSICAL AND PSYCHOLOGICAL THERAPEUTIC INTERVENTIONS

There are a growing number of studies which have explored the therapeutic effects of meditation, stress management, prayer, and other related interventions for various psychological and physical disorders including anxiety, hypertension, heart disease, and cancer (Levin and Vanderpool, 1989; Leserman et al., 1989; Kabat-Zinn et al., 1992; Levin, 1994; Miller et al., 1995; Massion et al., 1995; Schneider et al., 1995; Zamarra et al., 1996). Performing high quality studies is essential to demonstrating the relationship between spirituality and health especially in light of the various criticisms that have been raised regarding methodological issues with these early studies (Sloan et al., 1999; Sloan and Bagiella, 2002). Such studies also provide insight into the longer term effects of spiritual practices and experiences.

## THEOLOGICAL AND EPISTEMOLOGICAL IMPLICATIONS

In considering the neuroscientific approach to religious and spiritual phenomena, one can ponder whether theological and epistemological issues can actually be addressed, sometimes referred to as “neurotheology” (Newberg, 2010). For example, brain correlates may help explain certain elements of spiritual practices. However, a biological correlate does not necessarily negate an actual spiritual component. Even situations in which religious states are induced by pharmacological agents does not necessarily detract from the spiritual nature of these states for the individual. For example, Shamanic practices in which various substances are ingested to aid in the spiritual journey are not viewed as less real or less spiritual by the participants because of the use of these exogenous substances. However, it is also important to recognize that religious practices do not confer an epistemically privileged position from which to ascertain “the reality.” Either way, this field of research may ultimately tread upon some fascinating, and problematic, philosophical issues. Such issues as they pertain to the experience of reality and the realness of religious phenomena should at least be kept in mind by researchers as this field expands.

## CONCLUSION

While the neuroscientific study of religious and spiritual phenomena has advanced substantially since some of the initial studies performed over 30 years ago, this field of research is still in its nascent stages. There are many unique methodological challenges facing this field in addition to the usual barriers of funding and academic stature. However, pursuing such projects may pay large dividends both for science and spiritual disciplines. From the religious perspective, such studies may help toward a better understanding of the human experience of spirituality and religion. From the scientific perspective, such research may help elucidate the complex workings of the human brain as well as the overall relationship between brain states and body physiology. Ultimately, if the methodological challenges can be met, studies of spiritual practices and their associated experiences could provide important knowledge for linking our scientific and spiritual pursuits.

## Conflict of Interest Statement

The author declares that the research was conducted in the absence of any commercial or financial relationships that could be construed as a potential conflict of interest.
